# Supplementary material for: Multi-Modal Compositional Analysis of Layered Paint Chips of Automobiles by the Combined Application of ATR-FTIR Imaging, Raman Microspectrometry, and SEM/EDX
Source: Molecules. 2019 Apr 8;24(7):1381. doi: 10.3390/molecules24071381 (PMC6479518; doi:10.3390/molecules24071381)

Supplementary Information for publication

Multi-modal compositional analysis of layered paint chips of automobiles by the combined application of ATR-FTIR imaging, Raman microspectrometry, and SEM/EDX

Md Abdul Malek[a], Takashi Nakazawa[b], Hyun-Woo Kang[a], Kouichi Tsuji[b], and Chul-Un Ro[a,*]

a Department of Chemistry, Inha University, Incheon 22212, Korea

b Graduate School of Engineering, Osaka City University, Osaka 558-8585, Japan

* Correspondence to: C.-U. Ro, Department of Chemistry, Inha University, Incheon 22212, Korea. Phone: +82 10 6381 1400 and Fax: +82 32 867 5604. E-mail: curo@inha.ac.kr

A summary of content of the supplementary information:

- Number of pages: pp. S1- S16
- Figures S1 to S15: pp. S2 – S16.

Figure S1. Representative ATR-FTIR spectra of layers #1, #2, #4, and #5 of sample A in comparison with two library spectra showing the best match. Peak notations are ↓: alkyd, ♥: melamine, and ▽: PAP.

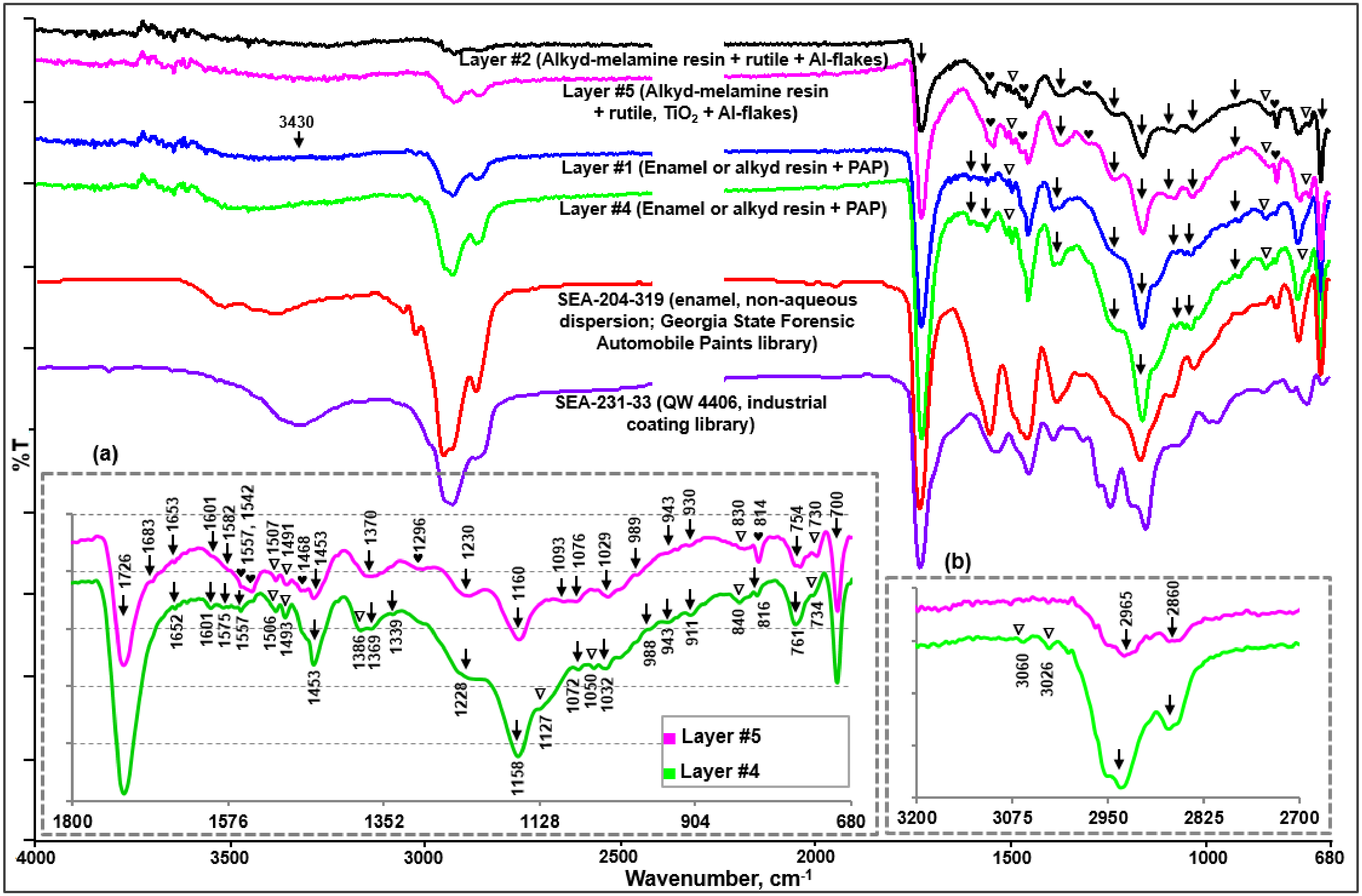

Figure S2. Representative Raman spectra of layers #2, #3, #6 and #7 of sample A (magnified in an inset over 750 – 1800 cm^{-1}) compared with that of standard rutile, anatase, and kaolinite. Peak notations are ★: rutile (TiO_2); ▽: anatase (TiO_2)

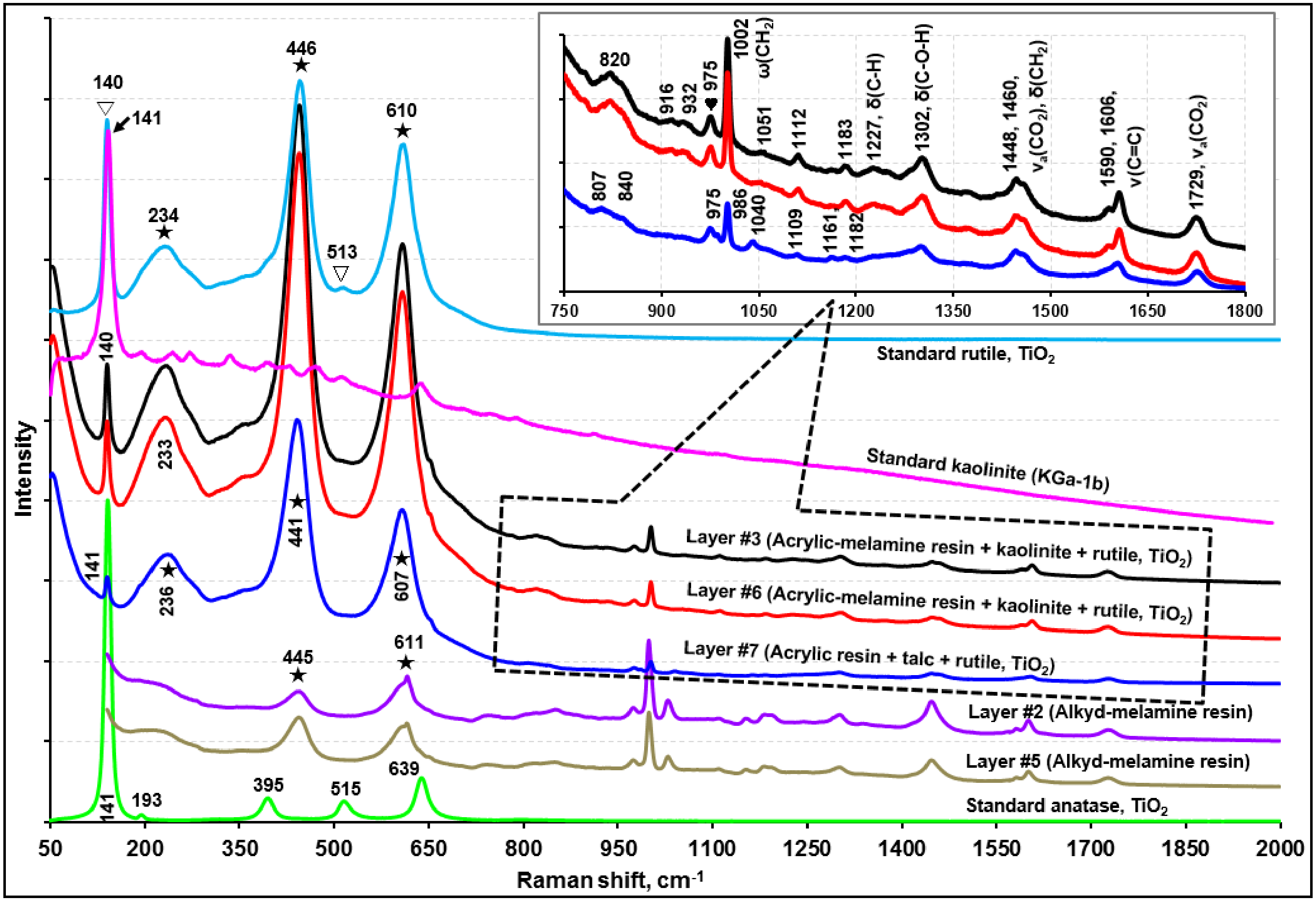

Figure S3. Representative ATR-FTIR spectra of layers #3, #6, and #7 of sample A in comparison with two library spectra showing the best match. The peak notations are ◊ : acrylic, ♥: melamine, ♦: kaolinite, and ★: TiO_2.

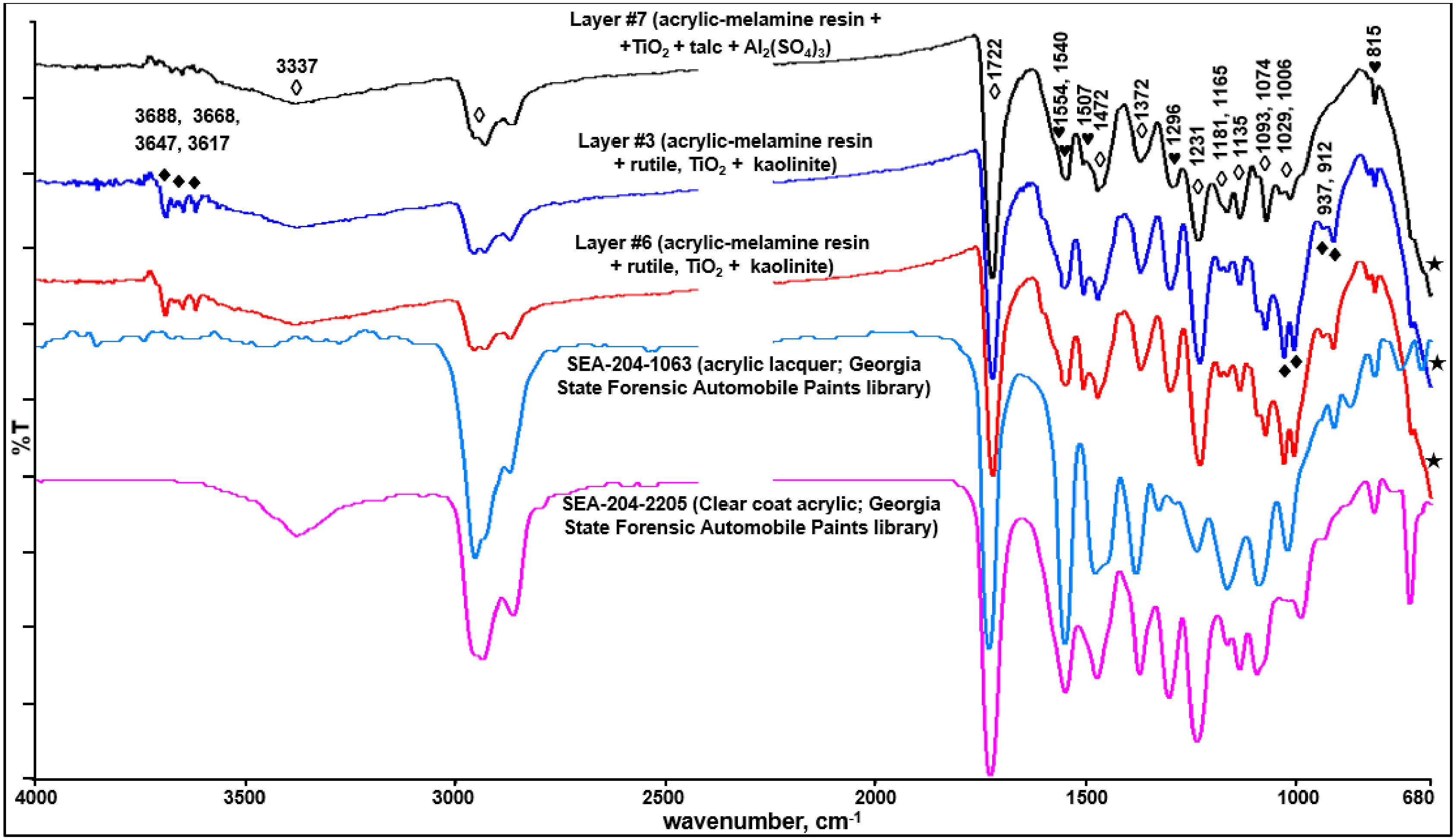

Figure S4. ATR-FTIR spectra of layers #3, #6, and #7 of sample A and standard kaolinite, talc, and rutile (TiO_2). The peak notations are ◊ : acrylic, ♥: melamine, ♦: kaolinite, ∗: TiO_2, ▲: talc, and △: SO_4^{2-}.

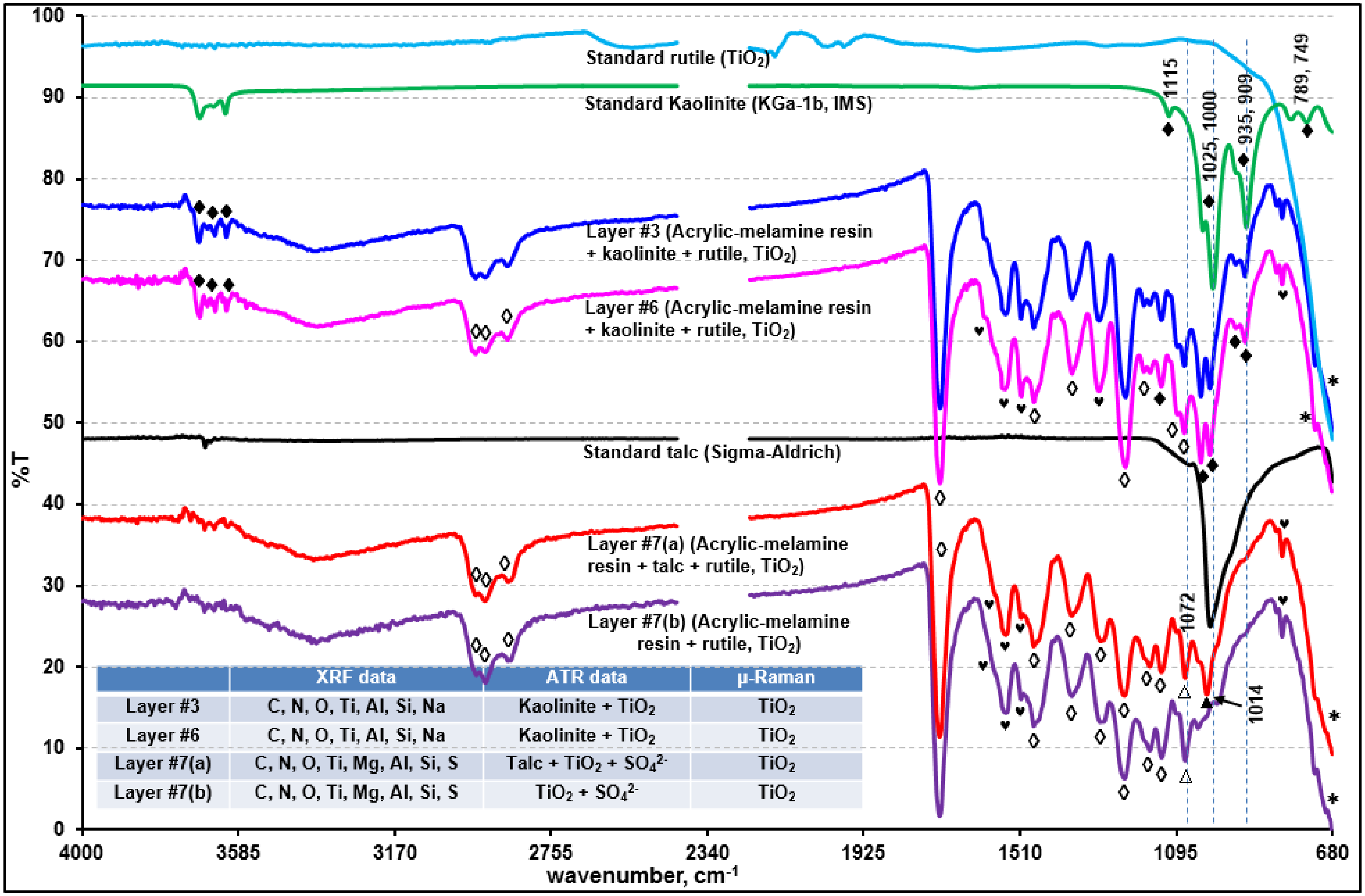

	XRF data	ATR data	μ-Raman
Layer #3	C, N, O, Ti, Al, Si, Na	Kaolinite + TiO_2	TiO_2
Layer #6	C, N, O, Ti, Al, Si, Na	Kaolinite + TiO_2	TiO_2
Layer #7(a)	C, N, O, Ti, Mg, Al, Si, S	Talc + TiO_2 + SO_4^{2-}	TiO_2
Layer #7(b)	C, N, O, Ti, Mg, Al, Si, S	TiO_2 + SO_4^{2-}	TiO_2

Figure S5. (a) Two pairs of X-ray spectra for layers #3 and #6 of sample A, showing that Ti peak intensities increase as Al and Si decrease and vice versa and (b)-(d) X-ray spectra obtained at various locations in layers #7-#9 of sample A.

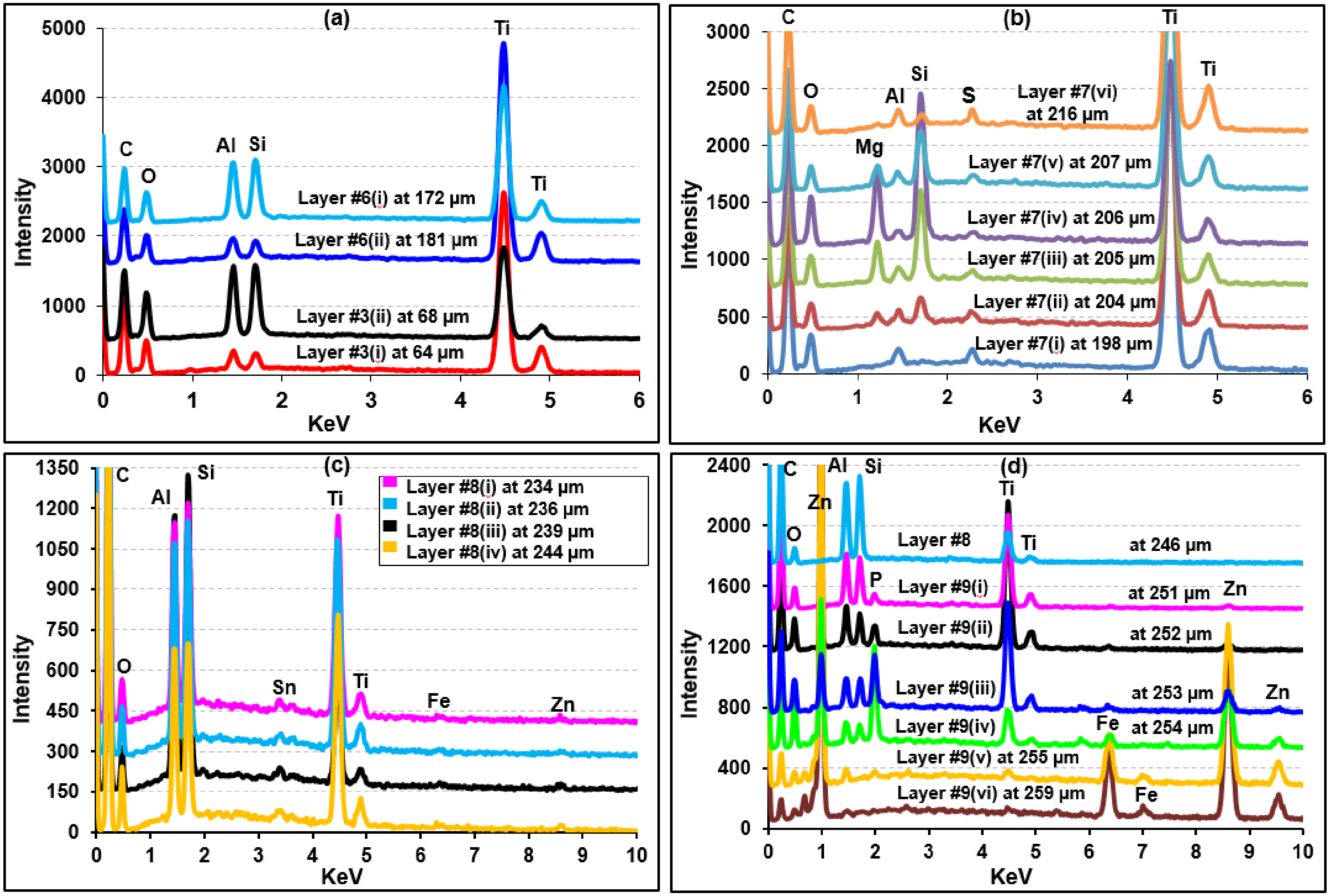

Figure S6. Representative ATR-FTIR spectra of layers #8 and #9 of sample A in comparison with a spectrum of the resin mold used for the cross-section preparation and two library spectra of epoxy resins with and without carbonyl peak at ~1730 cm^{-1}. [*DGEBA: Diglycidyl ether of bisphenol-A]

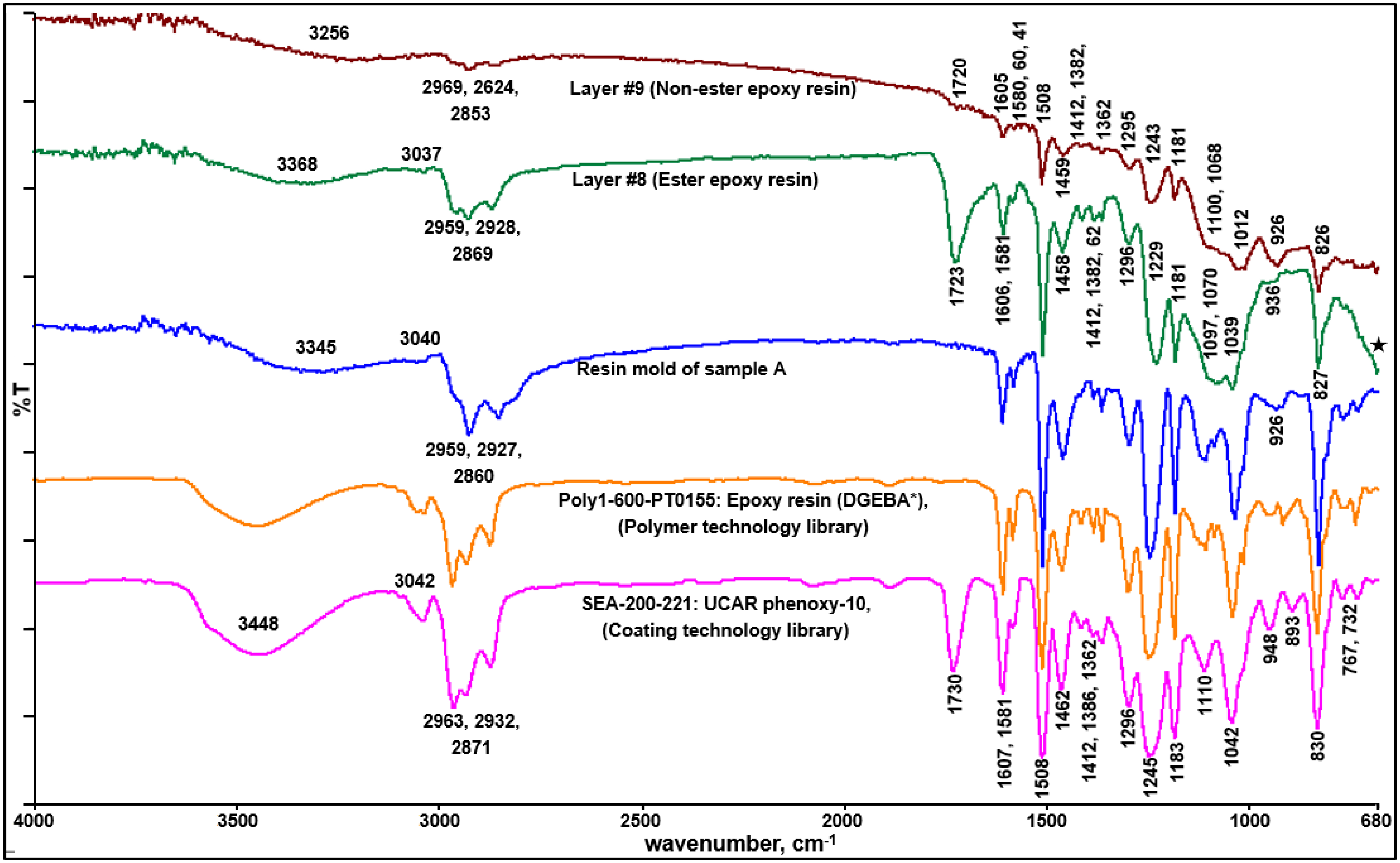

Figure S7. ATR-FTIR spectra of layers #8 and #9 of sample A in comparison with those of the resin mold used for cross-section preparation and standard kaolinite, pyrophyllite, and $Zn_3(PO_4)_2$.

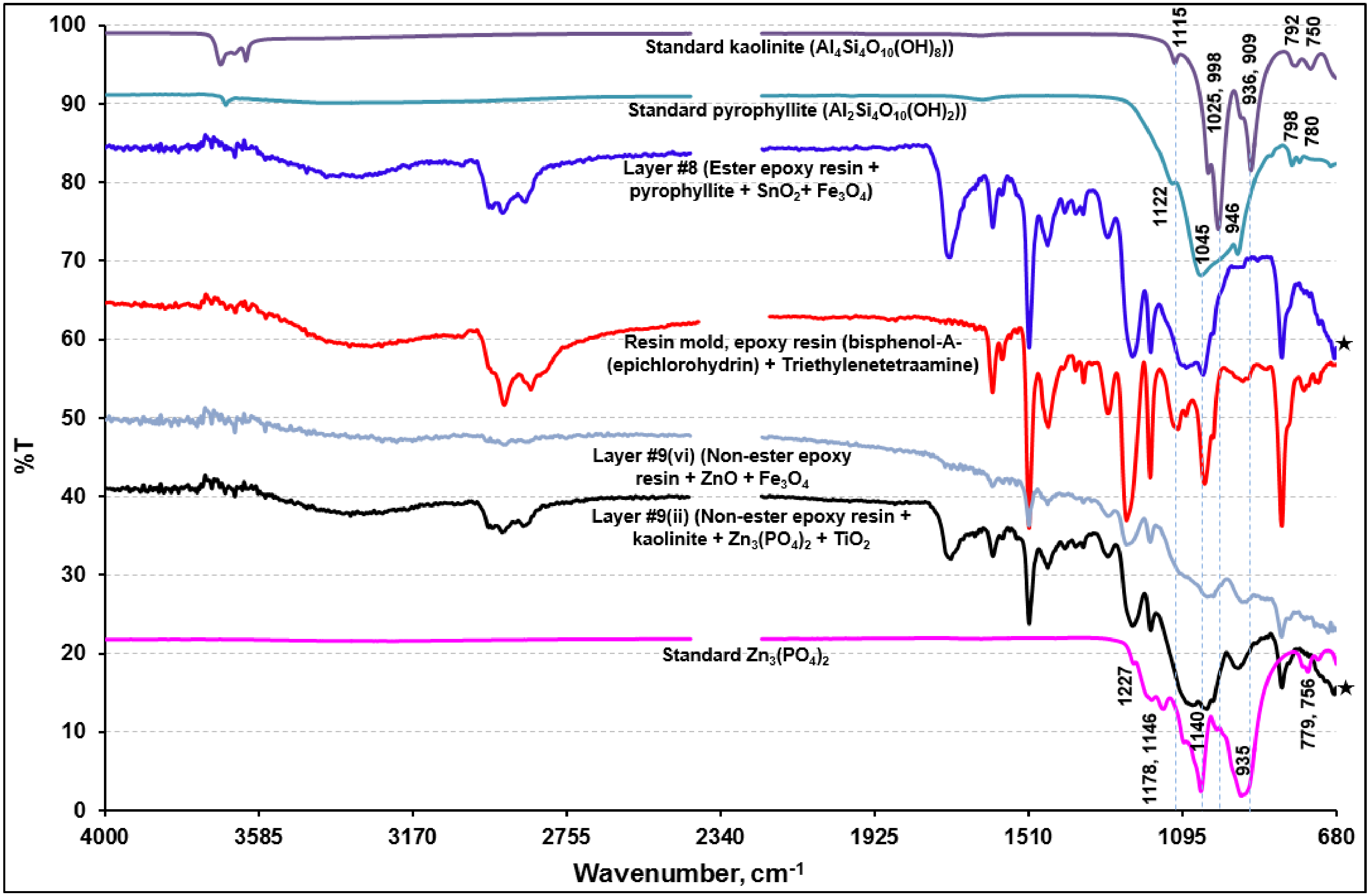

Figure S8. Representative Raman spectra of layers #8 and #9 of sample A in comparison with standard Fe_3O_4.

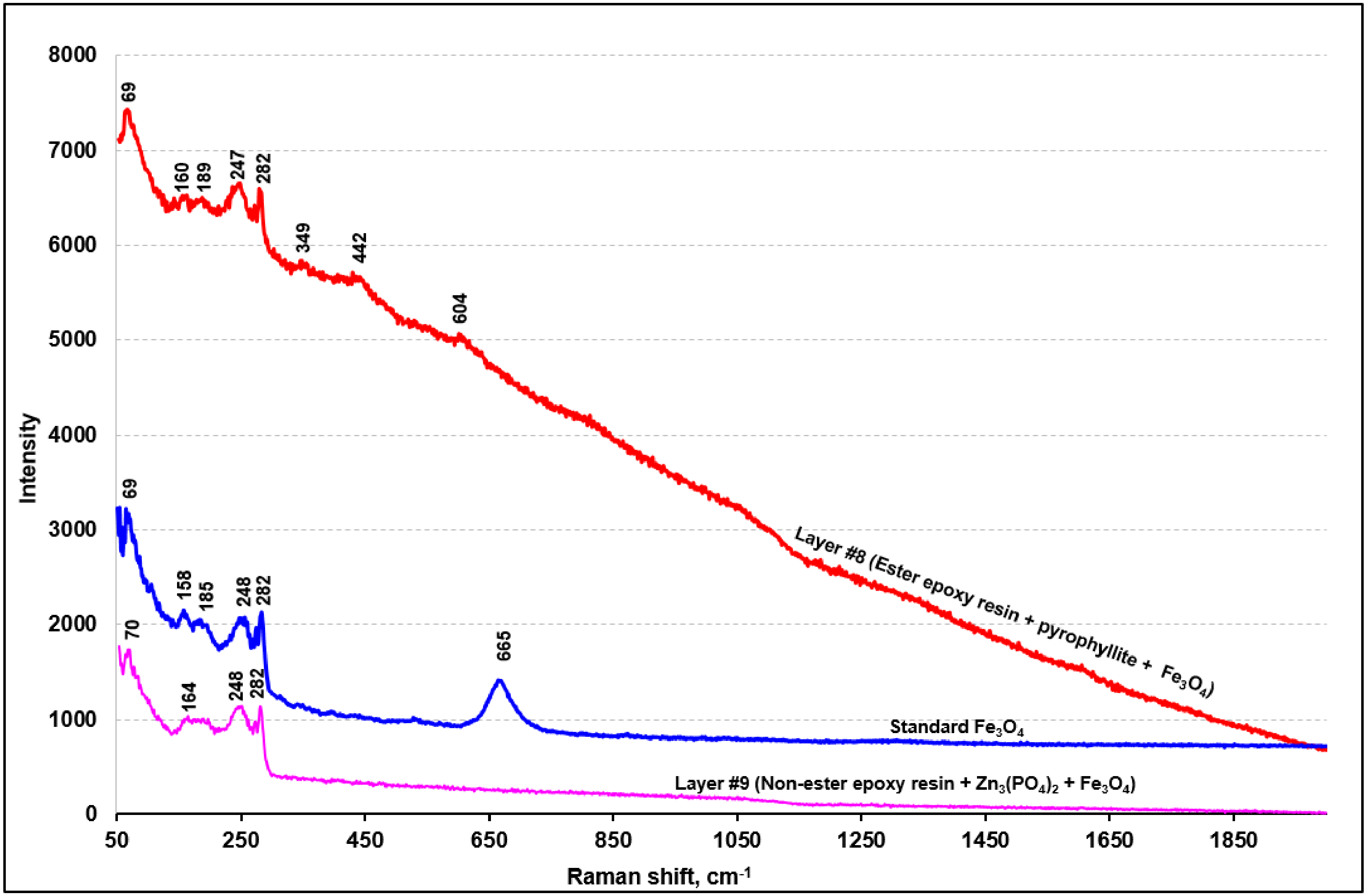

Figure S9. Plot of the baseline corrected ATR-FTIR spectra at successive pixels on a line across the boundary between layer #8 and #9 of sample A. ATR-FTIR spectra of standard kaolinite, pyrophyllite, and $Zn_3(PO_4)_2$ are included for comparison.

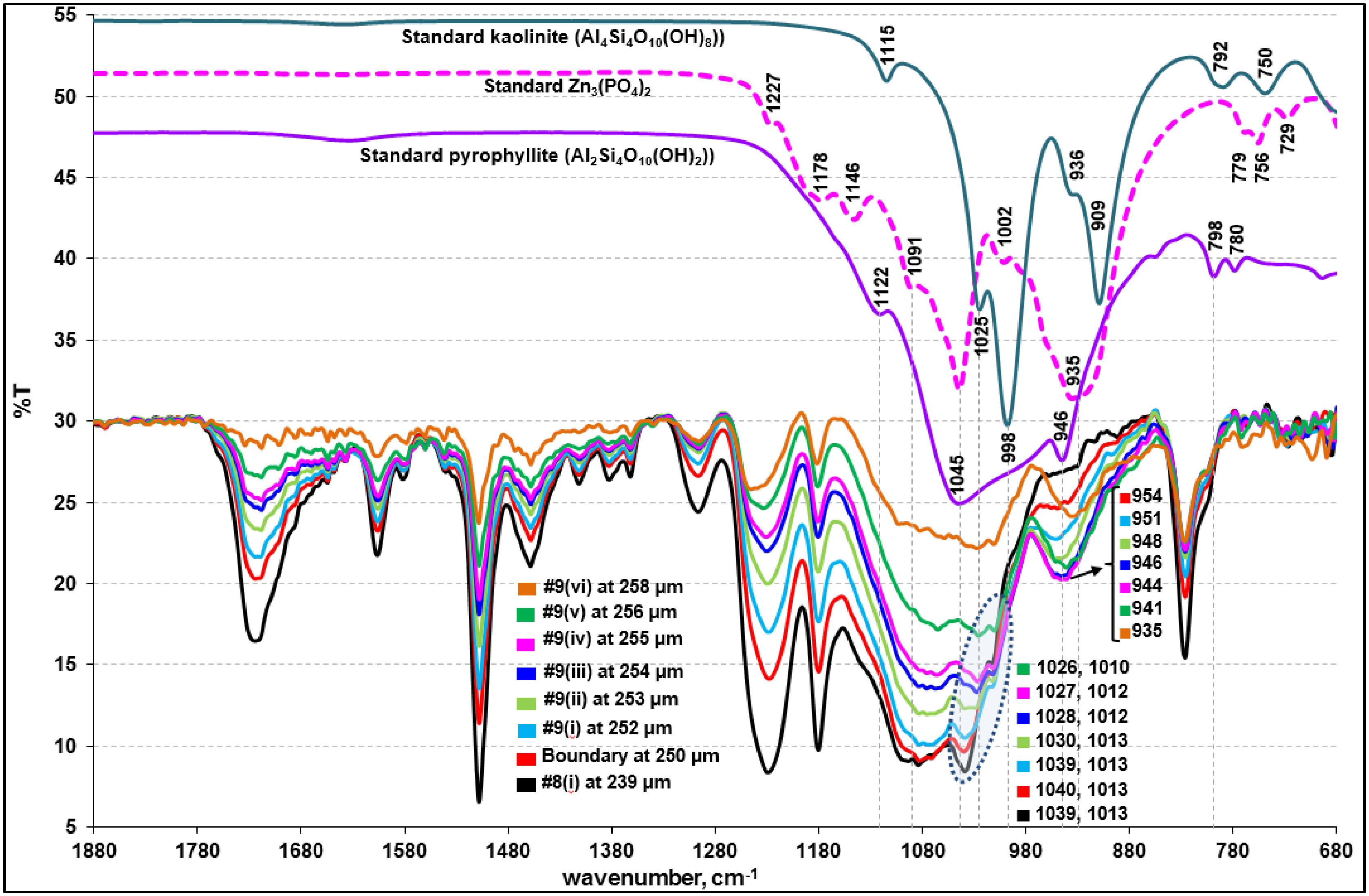

Figure S10. Representative ATR-FTIR spectra of layer #1 of sample B in comparison with a spectrum of the resin mold used for cross-section preparation, standard kaolinite, and two library spectra showing the best match. The peak notations are ↓: epoxy, ♦: kaolinite, and ★: rutile (TiO_2).

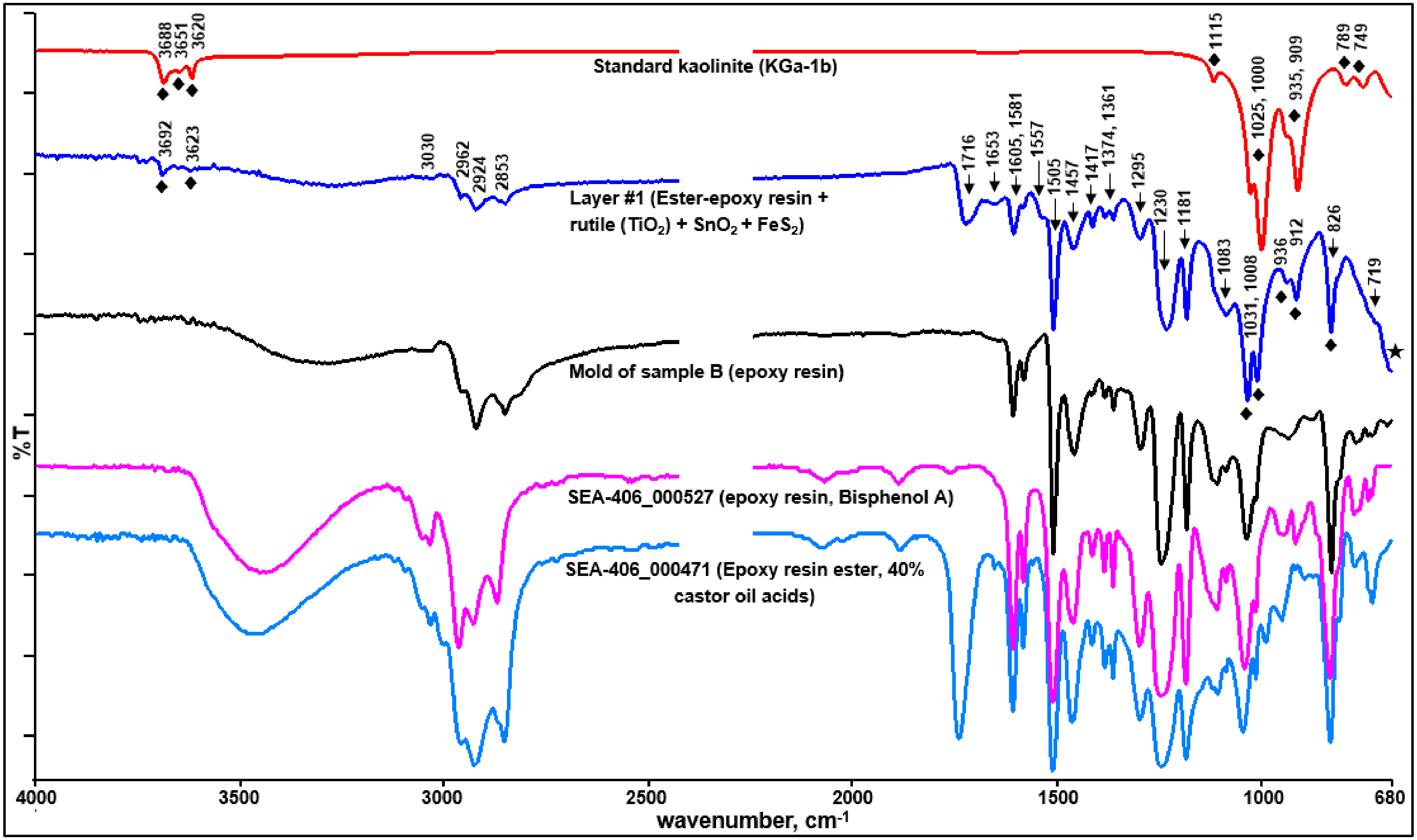

Figure S11. Representative ATR-FTIR spectra of layers #2, #3, and #4 of sample B in comparison with a spectrum of standard $BaSO_4$ and three library spectra showing the best match. The peak notations are ↓: alkyd, ♥: melamine, ★: TiO_2, and S: SO_4^{2-} .

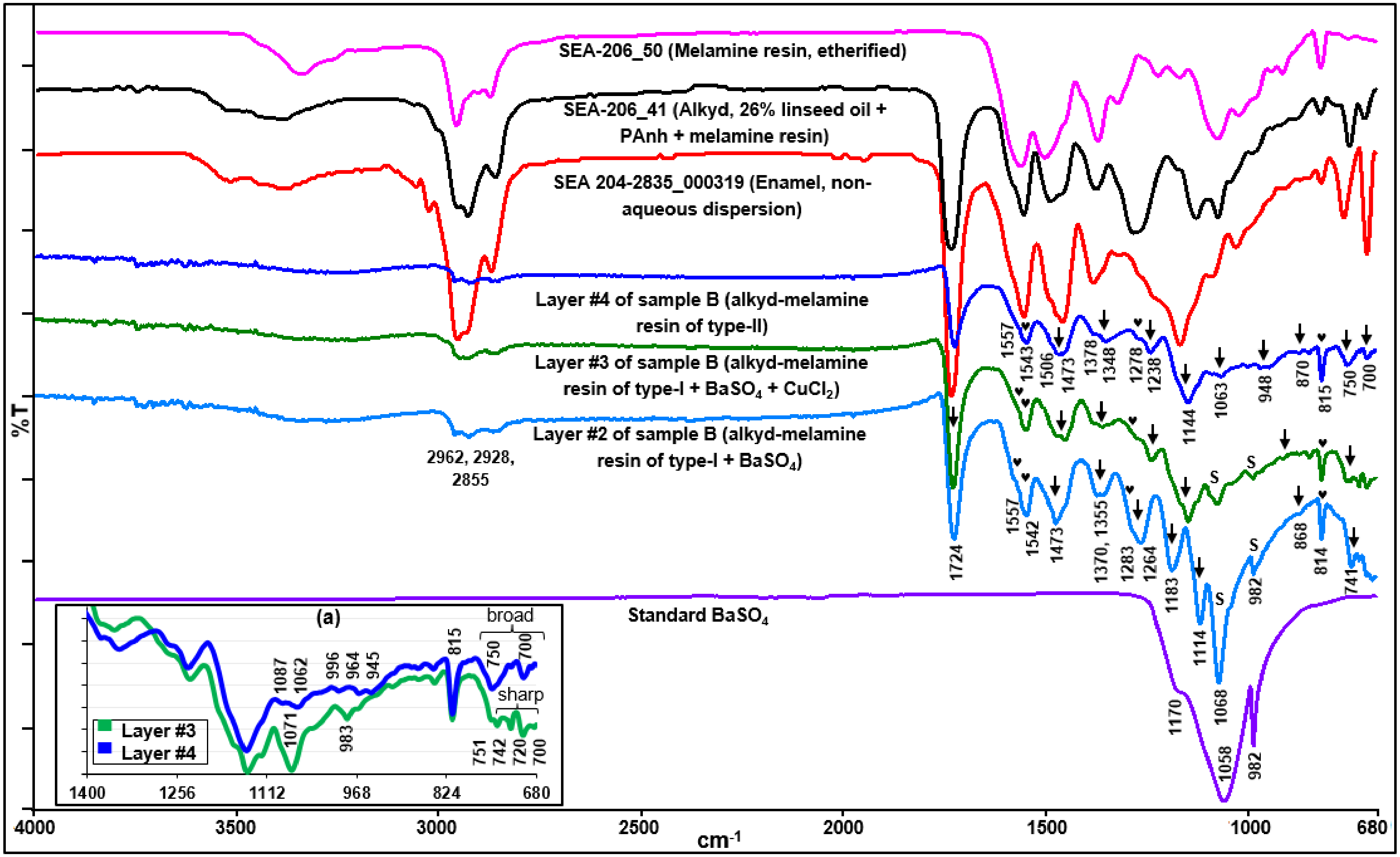

Figure S12. Representative ATR-FTIR spectrum of layer #5 of sample B and three library spectra showing the best match.

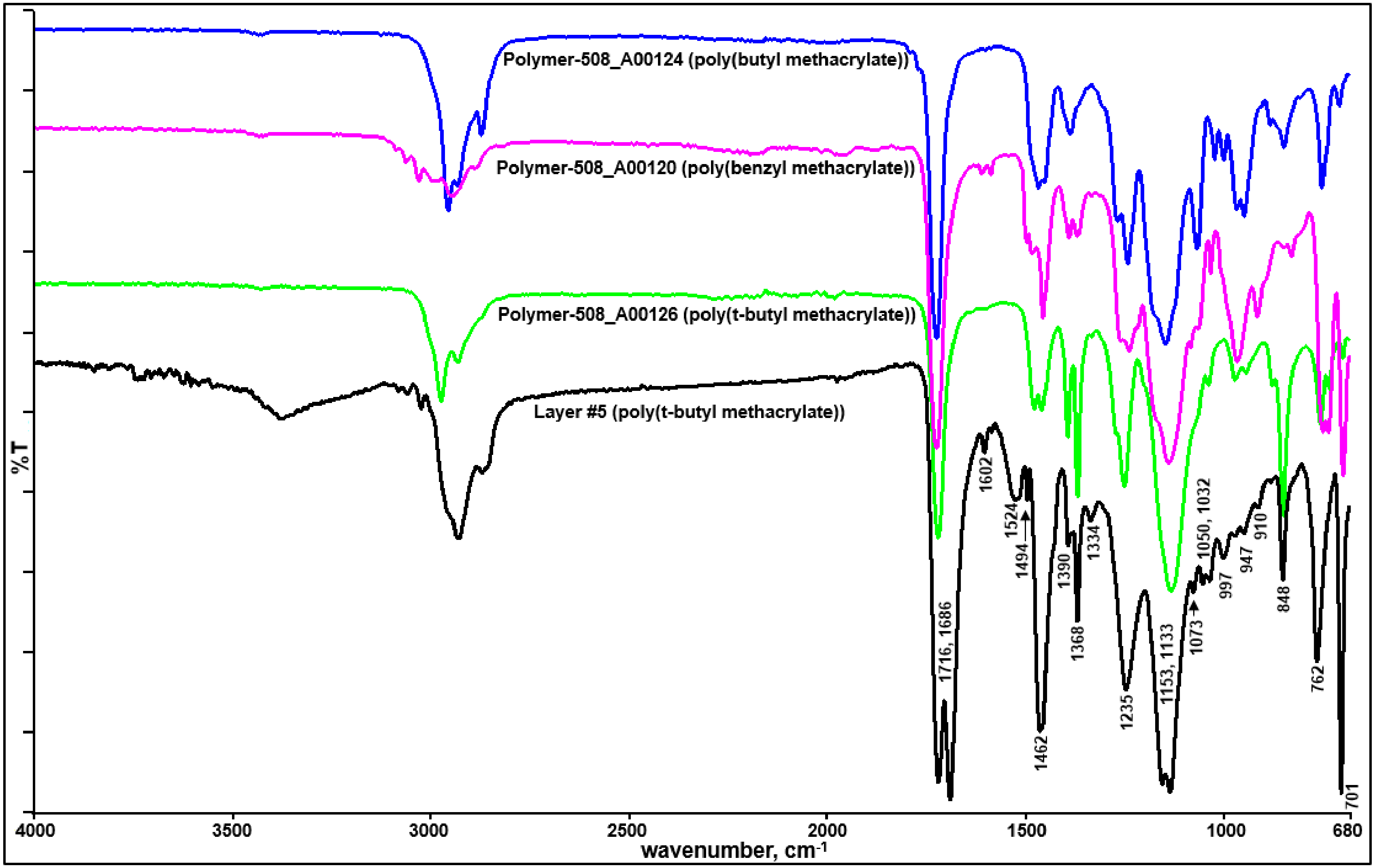

Figure S13. Representative ATR-FTIR spectra of layers #1 and #2 of sample C and two library spectra showing the best match.

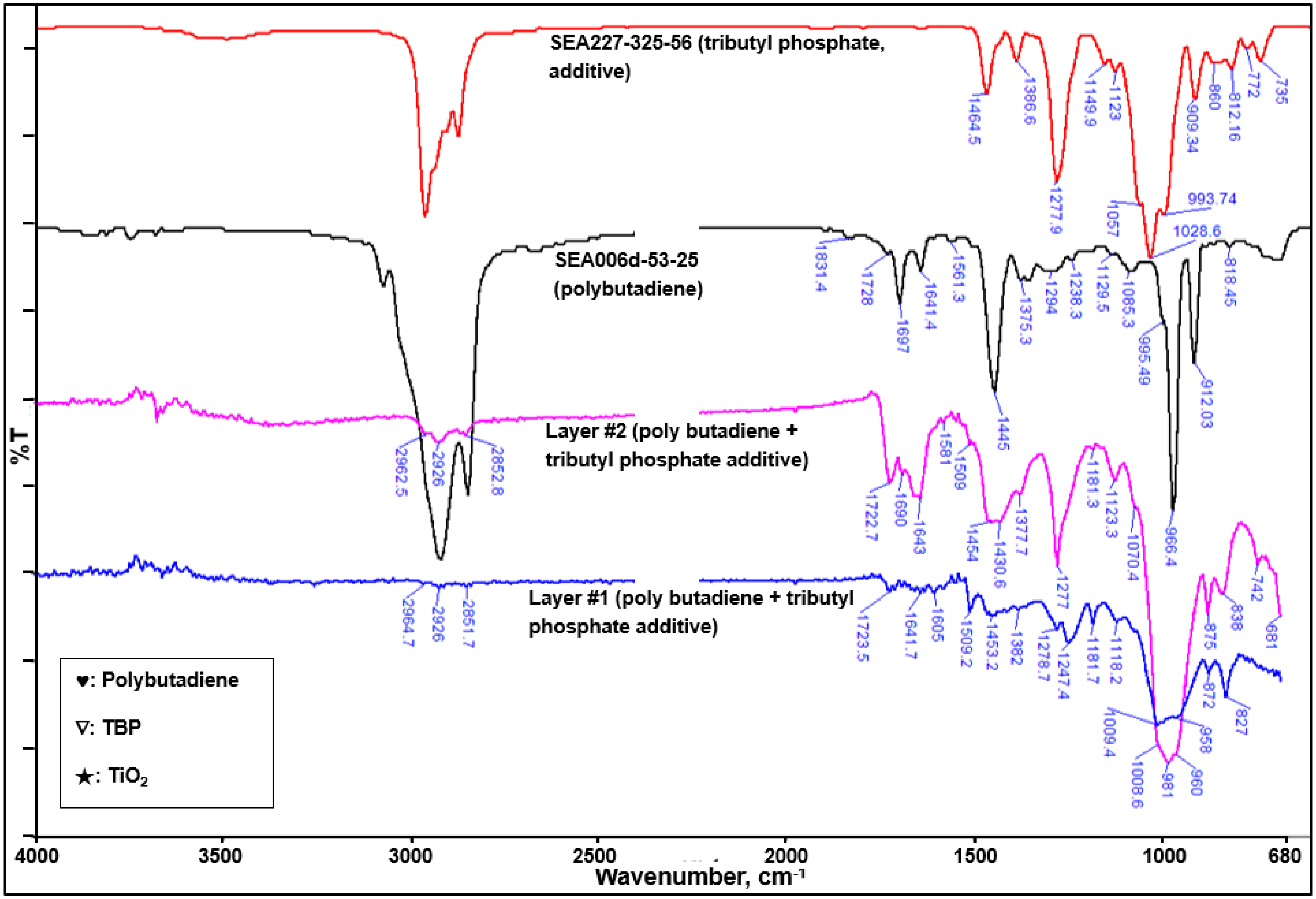

Figure S14. ATR-FTIR spectra at different locations within layers #1 and #2 of sample C in comparison with those of standard talc and $CaCO_3$. The peak notations are ↓: poly butadiene, ♦: TBP, ★: rutile (TiO_2), C: $CaCO_3$, and T: talc.

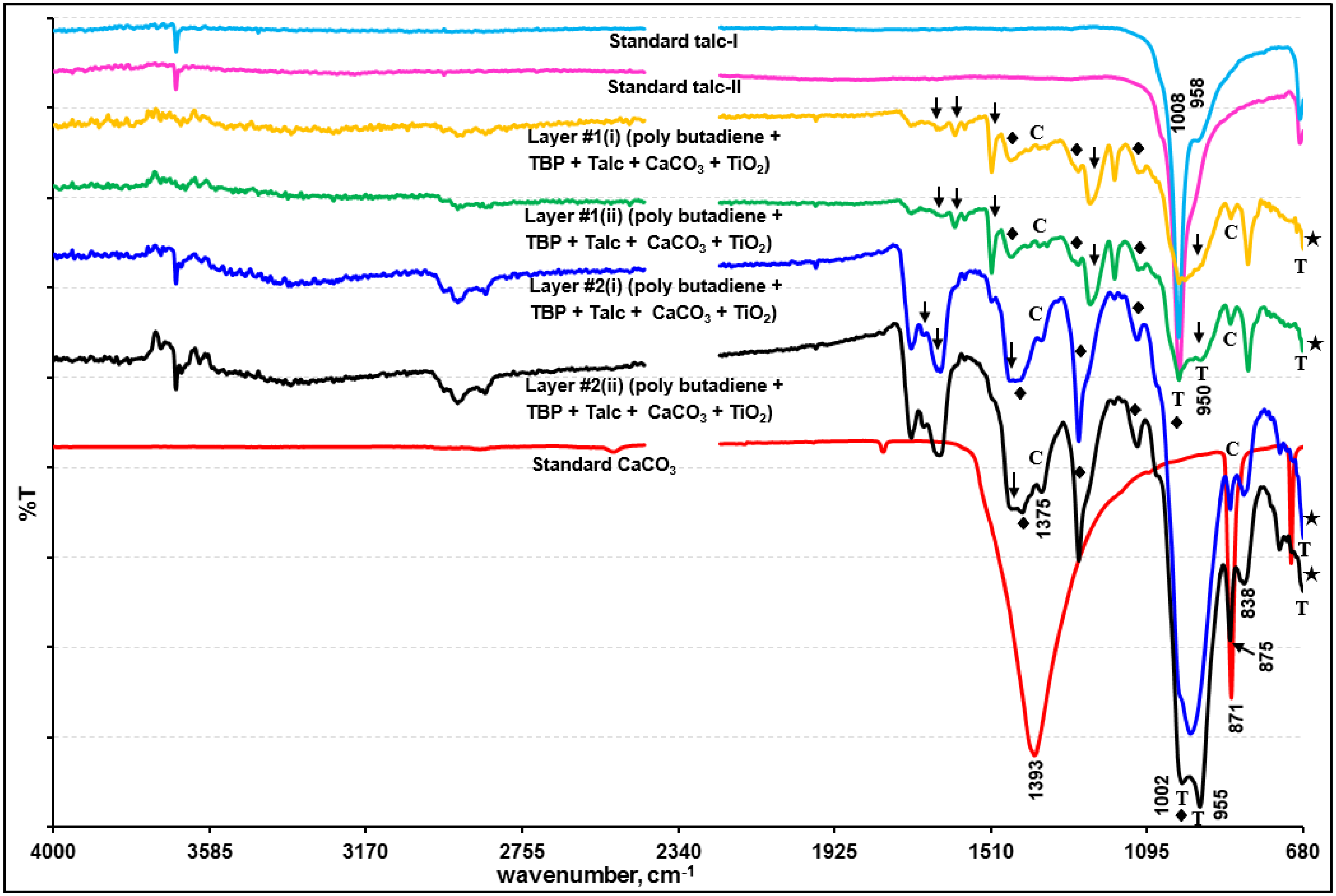

Figure S15. Representative ATR-FTIR spectra of layers #3 and #4 of sample C and one library spectrum showing the best match.

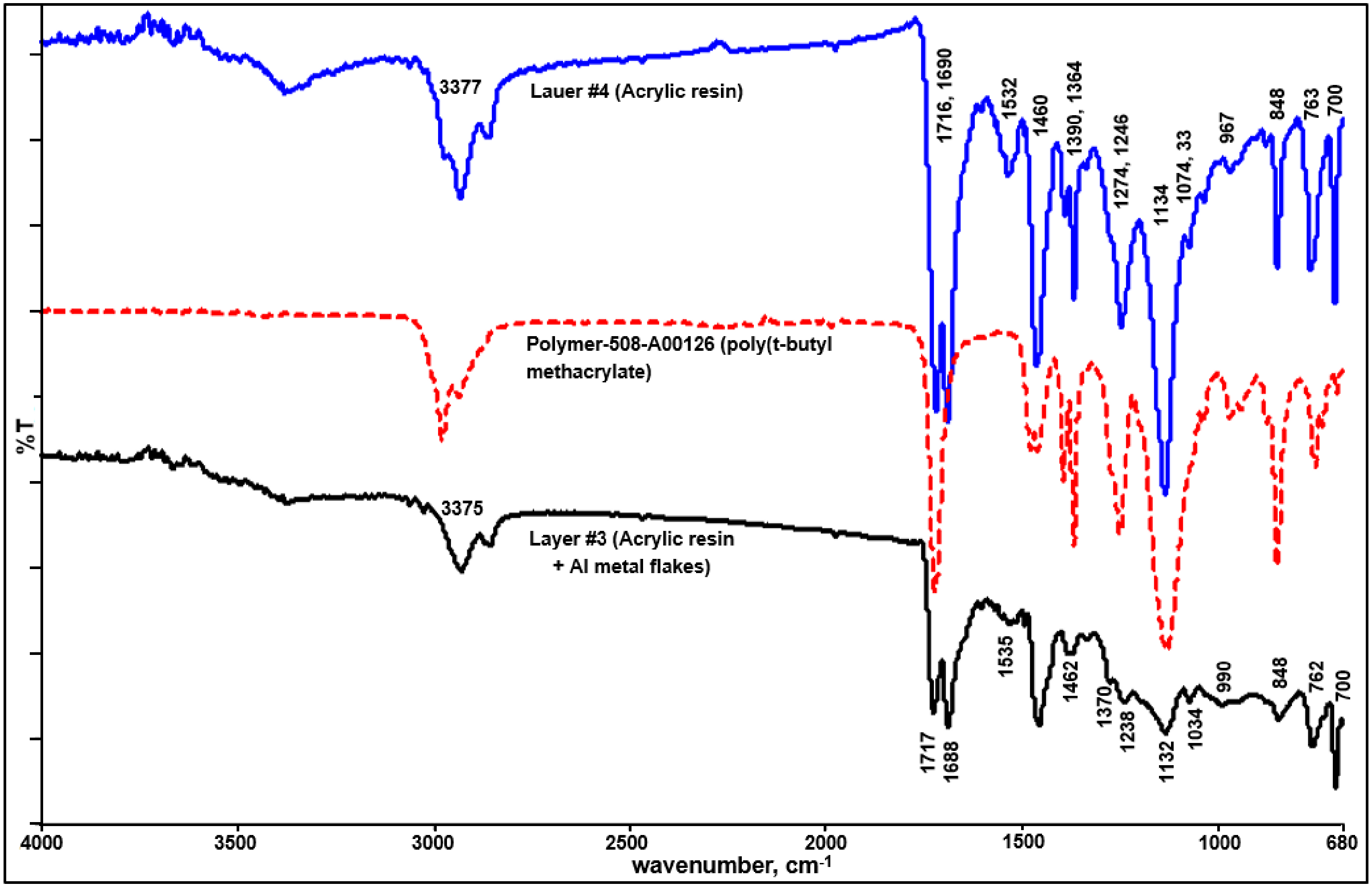

Supplement: Supplementary file 1 [file molecules-24-01381-s001.pdf]
